# Hybrid Growth Modes of PbSe Nanocrystals with Oriented Attachment and Grain Boundary Migration

**DOI:** 10.1002/advs.201802202

**Published:** 2019-02-28

**Authors:** Feng Cheng, Linyuan Lian, Luying Li, Jiangyu Rao, Chen Li, Tianyu Qi, Zhi Zhang, Jianbing Zhang, Yihua Gao

**Affiliations:** ^1^ Center for Nanoscale Characterization & Devices Wuhan National Laboratory for Optoelectronics Huazhong University of Science and Technology 1037 Luoyu Road Wuhan Hubei 430074 China; ^2^ Engineering Research Center for Functional Ceramics Ministry of Education, School of Optical and Electronic Information Huazhong University of Science and Technology 1037 Luoyu Road Wuhan Hubei 430074 China; ^3^ School of Physics Huazhong University of Science and Technology 1037 Luoyu Road Wuhan Hubei 430074 China

**Keywords:** atoms reconfiguration, grain boundary migration, oriented attachment, PbSe nanocrystals

## Abstract

The growth of nanocrystals has widely been researched recently through an in situ high‐resolution transmission electron microscopy technique, which reveals the process of morphological and structural evolutions. For nanocrystals, the underlying growth modes are mostly determined by growth environment and crystal morphology. Here, the direct growth process of the PbSe nanocrystals via controlling the temperature is clearly observed. The results show that the PbSe nanocrystals start growth following oriented attachment growth mode, and then change to growth with grain boundary migration at moderate temperature as the heat activated nanocrystals gather together with decreased degree of freedom for crystal rotation. During the grain boundary migration, the smaller nanocrystals are inclined to be assimilated by larger ones through interfacial atom reconfigurations, which are observed to take place through strain mediated atom migration. The growth mode changes in different growth states with a hybrid growth mode of oriented attachment and grain boundary migration during the whole growth process.

## 1 Introduction

Nanocrystals (NCs), also known as quantum dots, have attracted great attention due to their remarkable performances in optoelectronic applications such as light‐emitting diodes,^1^, ^2^, ^3^ photodetectors,^4^, ^5^, ^6^ and photovoltaic devices.^7^, ^8^ The NCs‐based optoelectronic devices show distinguished features including compact size, broad excitation band, large absorption cross‐section, high fluorescence quantum yield, superior photostability, and solution processability. Their unique nanostructures are also extensively studied as to structural crystal growth, characterization, phase transition, etc.^9^, ^10^ Colloidal NCs are considered as perfect pieces of their bulk counterparts since they are normally free of non‐equilibrium crystal defects.^11^, ^12^ The formation of 2D nanoplates by atomic connection of smaller crystals is an important growth process in morphology,^13^, ^14^ which also finds great applications in 2D optoelectronic devices.^15^, ^16^ Since NCs with different growth modes normally show different physical and chemical properties, detailed investigations on NCs growth processes may help better understand the phase transformation and the underlying growth mechanisms.^17^

As a common growth mode in NCs, oriented attachment gains enormous attention, with wide applications in the preparation of metal and semiconductor NCs.^18^ Oriented attachment mainly consists of the following three steps: (1) adjacent particles spontaneously self‐organize so that they share a common crystallographic orientation; (2) the particles fuse to create a planar coherent interface; (3) the primary particles are perfectly aligned during growth,^14^, ^19^ as implied by the diffraction of a single crystal structure. It is reported that the gold nanowires are formed by oriented attachment of gold nanoparticles with different geometries.^20^ CdSe with different structures are prepared under different growth conditions according to oriented attachment growth mode, which results in different electrical properties.^21^, ^22^, ^23^

The oriented attachment is realized by attaching existing dot‐shaped NCs along a given crystal orientation.^24^ The attachment is accompanied by the rotation of the adjacent NCs which require enough spaces to provide more degrees of freedom.^25^ It is reported that the growth conditions, such as growth temperature and time, will directly affect the material growth process.^26^ For NCs growth, the growing crystals show different geometries at different stages. Especially at high temperatures, the NCs are highly activated, and the contact between different crystals are inclined to be random, which will directly reduce the degree of freedom in 3D space, leading to a complicated material growth environment, and the corresponding growth mode would also be affected.

PbSe NC has excellent intrinsic optoelectronic properties: with the exciton Bohr radius of 46 nm, it exhibits strong quantum confinement effect;^27^ it can easily form epitaxial connections between well‐oriented proximal NCs (2D superlattices);^28^ it has high multiple exciton generation efficiency,^29^ and PbSe NC‐based solar cells have realized EQE of above 100%.^30^ PbSe NCs are normally reported to follow the oriented attachment growth mode, which show various geometrical structures and good stability.^31^, ^32^ The related phase transformation investigations such as heterojunction preparation and in situ growth are also reported.^33^, ^34^ While the recent reports on PbSe NCs growth mainly focus on the growth process in original state with regular shape and high degree of freedom, the growth of NCs with random orientations and complicated grain boundaries are rarely investigated, which could also be an important part of the whole growth process with different growth mechanism.

Besides the basic function of static structural characterization, transmission electron microscope (TEM) can also provide various stimulations (“heat” in the current case), and the dynamic process of phase transitions can be recorded at atomic resolution, which is beneficial for further understanding of the underlying growth mechanisms.^35^, ^36^ In this study, PbSe NCs are successfully synthesized, and the high‐resolution transmission electron microscopy (HRTEM) with in situ heating techniques are used to reveal the evolution of NC structures during the growth process. By controlling the growth temperature and growth time, the PbSe NCs with different geometries are clearly observed, based on which the underlying growth mechanisms at different growth stages are revealed.

## 2 Results and Discussion

The structural characterization and in situ heating experiment were carried out using a FEI Titan G^2^ 60–300 transmission electron microscope operating at 300 kV. The in situ heating of PbSe NCs was performed using DENS solutions‐DH30‐4M‐JU, double‐tilt heating/biasing holder (chip: wildfire DS2049‐W3‐R1). The PbSe nanocrystals were dispersed in tetrachloroethylene (TCE) solvent and then dropped on the chips compatible with the heating stage for TEM observations. During the in situ observation, the temperatures were set to fixed values to keep the sample in a relatively stable state, sequential HRTEM images were recorded by a CCD camera to track the structural evolutions during heating.

**Figure 1:**
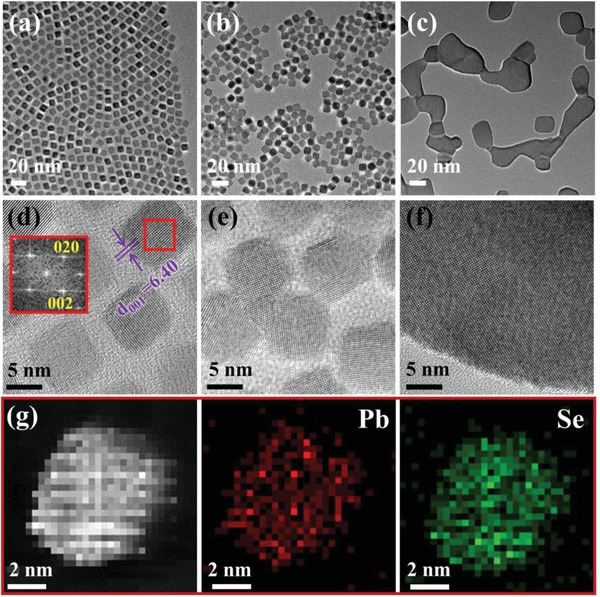
Morphologies, crystal structures and chemical information of PbSe NCs. a) TEM image of PbSe NCs at room temperature. b) PbSe NCs with random orientations after heating for 1 h at 180 °C. c) The comparatively large PbSe crystal agglomerated by NCs of smaller size after heating for 1 h at 380 °C. d–f) The HRTEM images corresponding to the same experimental conditions as in insets (a)–(c), respectively. g) HAADF image of single PbSe NC, and the corresponding elemental maps of Pb and Se.

**Figure** **1**a–c is TEM images of PbSe NCs at various temperatures and the corresponding HRTEM images are shown in Figure 1d–f. The as‐prepared NCs with lateral size of ≈8 nm are uniform cubes of face‐centered cubic (fcc) structure with high crystal quality and the NCs appear to be oriented similarly. The lattice constant (*a* ≈ 0.640 nm) of the PbSe NC, measured via the Fast Fourier Transform (FFT) of the HRTEM image, is larger than the bulk counterpart (*a* = 0.612 nm), which may be attributed to the quantum size effect with obvious strain relaxation.^37^ The elemental maps of Pb and Se of the NC in Figure 1d labeled by a red box are presented in respective colors in Figure 1g, which show uniform distributions of both elements. As the temperature increases, the NCs tend to merge with adjacent ones and finally become a crystal of much larger size (Figure 1f). Figure 1e shows that the NCs are activated during continuous heating process, the orientation of the NCs become random and various interfaces are formed at the boundaries of neighboring NCs. This transitional state of crystal growth would bridge the initial (Figure 1a,d) and final states (Figure 1c,f) of the whole heating process, and provide better understanding of temperature dependence of the morphologies and atomic structures of PbSe NCs.

**Figure 2:**
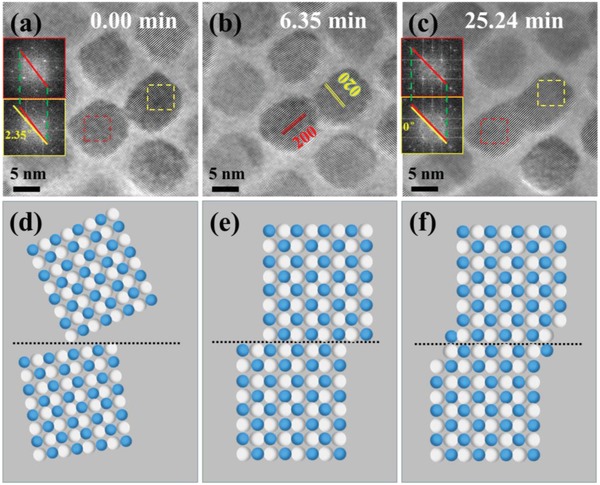
The evolution of the PbSe NCs under continuous heating at 180 °C. a–c) The HRTEM images of the PbSe NCs with different growth time at 180 °C. d–f) The sketches of the evolution of PbSe NCs corresponding to insets (a)–(c).

Movie S1 (Supporting Information) is the evolution of PbSe NCs under continuous heating at temperature of 180 °C, and **Figure** **2**a–c are the corresponding snapshots. Two NCs are connected with each other via their (010) facets, and the corresponding FFT of the NCs in Figure 2a indicates a misorientation angle of about 2.35° between them, which leads to slight bending of the crystal lattices at the neck of the interfacial region. With the prolonged heating time, the neck becomes more apparent (Figure 2b) as more atoms move towards the growing neck, a plateau appears around the neck region, which eventually turns into a rod‐like shaped interface. During the neck growth process, the NCs rotate slightly, which leads to the decrease of the lattice mismatch between the two NCs, and the misorientation angle approaches to 0° after 25 min of continuous heating at 180 °C, as labeled in Figure 2c. Thus, the adjacent NCs merge into bigger crystal through crystal rotation and side sliding, as shown in the corresponding sketches of Figure 2d–f, which are well matched with the growth mode of oriented attachment. The Movie S1 lasts for about 25 min, which shows almost no change after 20 min. The heating process continues for another 20 min (not shown in Movie S1 in the Supporting Information), and no obvious crystal growth is observed except for lattice deterioration due to prolonged electron beam irradiation. Further crystal growth may require higher temperature as the driving force rather than prolonged time.

van Huis et al. and Schapotschnikow et al. reported the oriented attachment mode as a direct attachment of planar surfaces via small, swift rotations, reorganization, and interfacial relaxation from both experimental and theoretical points of view.^38^, ^39^ In our case, in addition to the growth following oriented attachment mode at low temperature, the growth process is found to be quite different at relatively high temperature. Therefore, the dependences of the growth process on growth temperature, as well as the underlying mechanisms of the whole growth process are systematically studied.

**Figure 3:**
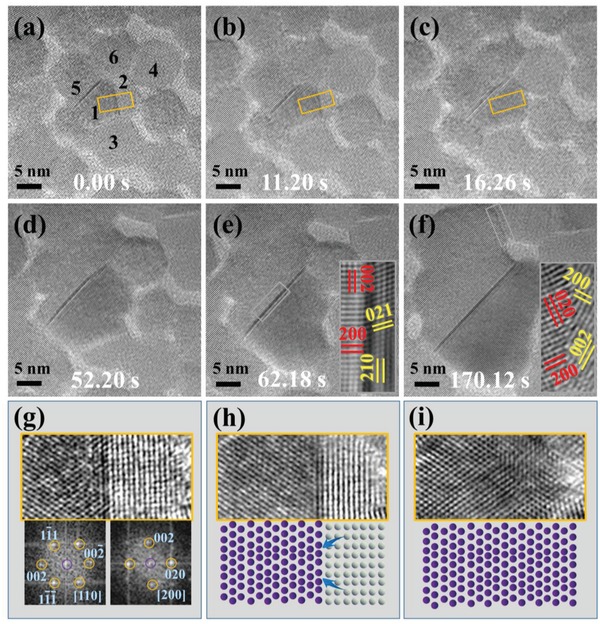
The grain boundary migrations of the PbSe NCs with low degree of freedom in 3D space. a–f) The HRTEM images of the PbSe NCs with different growth time at 280 °C, in which the number of NCs decreases and the NCs tend to merge into bigger crystals. g–i) The enlarged images correspond to the brown boxed regions in insets (a)–(c) showing the interfacial structures of the NCs during boundary migrations, FFT of both sides, and corresponding interfacial atomic models.

Different from the case in Figure 2, the NCs do not always touch only one NC. As **Figure** **3**a shows, after increasing the temperature to 280 °C, more NCs get together due to thermally activated migration of NCs on the substrate, some NCs contact with two or more neighboring NCs, and the interfaces between the NCs are random. NCs once in contact would further coalesce in order to reduce mutual surface energies,^40^, ^41^ leading to agglomerated crystals. Figure 3a–c displays the typical growth process of NC 1 and NC 2. Instead of showing any signs of rotation as in the case of oriented attachment growth mode, the growth starts through the reconfiguration of the atoms near the interface (Movie S2, Supporting Information), which corresponds well with the growth mode of grain boundary migration. It is noted that the current growth mode, as compared to oriented attachment, could be attributed to the reduction of freedom since the adjacent grains (NCs 3–6) may highly limit the lateral movements of NCs 1 and 2, which precludes the possibility of oriented attachment growth mode.

NC 1 assimilates NC 2 along the direction perpendicular to the interface. In order to analyze the interface structure, the corresponding enlarged images are shown in Figure 3g–i. The contact faces of NC 1 and NC 2 are (00$\bar{2}$) and (0$\bar{2}$0), respectively, which are equivalent for fcc structure with the same surface energy. The neighboring grains are misoriented by an angle of 45° in 3D space, which may lead to disordered atomic arrangements including Se–Se or Pb–Pb bonds. The corresponding density functional theory (DFT) calculations (Figure S1, Supporting Information) indicate that the bond energies for Se–Se and Pb–Pb are much higher than that of Pb–Se, which would result in much higher interface energy, lower stability, and easily reconstructed interface. Since the size of NC 1 is larger than NC 2, and the grain boundary moves to the NC 2 side, it is quite possible that the obvious size difference may guide the migration direction of the grain boundaries.

Similar to NC 1 and NC 2, most NCs follow the same growth mode: the grain boundaries migrate in certain directions, and the NCs of larger size assimilate those of relatively smaller size with vanishing grain boundary at last. However, in a special case, the grain boundary between NCs 1 and 5 clearly exists throughout the whole heating process, as shown in Figure 3a–f. At the contact interface, the (021) planes of NC 1 and (200) planes of NC 5 connect layer by layer, and the perfect lattice matching at the interface ensures low levels of strain fields and relatively stable interface.^41^ Since NCs 1 and 5 are of similar size, the boundary between them persists without obvious assimilation of either side.

Careful analysis of the grain boundary between NCs 1 and 5 reveals that the grain boundary extend upward as compared to the initial state during the process of crystal growth. As shown in Figure S2a–d (Supporting Information), the grain boundary of NCs 1 and 5 slightly moves rightward and integrates with the grain boundary between NCs 2 and 6. Then, the primary grain boundary continues to integrate with the grain boundary of NCs 4 and 6. On the other hand, the grain boundary of NCs 2 and 4, which is almost perpendicular to the primary grain boundary, is hardly affected. Thus, it seems that the primary stable grain boundary is inclined to combine with those grain boundaries of similar orientations.

The remaining grain boundary intersects the crystal of much larger size, as presented in Figure 3f, and stays still under prolonged heating at the same temperature. It is well known that for certain size of NCs, there exists a temperature limit that can trigger crystal growth,^42^ the large size of NCs may help improve the stability of material interface, and stop the migration of grain boundaries. Furthermore, the results also show that NC 3 and NC 4 with large volumes take much more time to finish their grain boundary migrations, as compared to NC 2 (NC 3 and NC 4 spend more than 30 s, NC 2 spends less than 20 s, respectively), which directly illustrate the size dependence of the total length of the grain boundary migration process.

In order to check if the growth mode is dependent on the original NC size, the PbSe NCs with smaller size of ≈5 nm are heated at 180 °C, and the results are shown in Figure S3 in the Supporting Information. These NCs are of spherical shape, and are relatively easier to accumulate than the larger NCs during continuous heating. It turns out that the region with only one grain boundary (region 1 in a black box) follows the oriented attachment growth mode, and the region contains several grain boundaries and less degree of freedom (region 2 in a red box) shows obvious grain boundary migration, which are almost the same as the case of larger NCs (8 nm).

**Figure 4:**
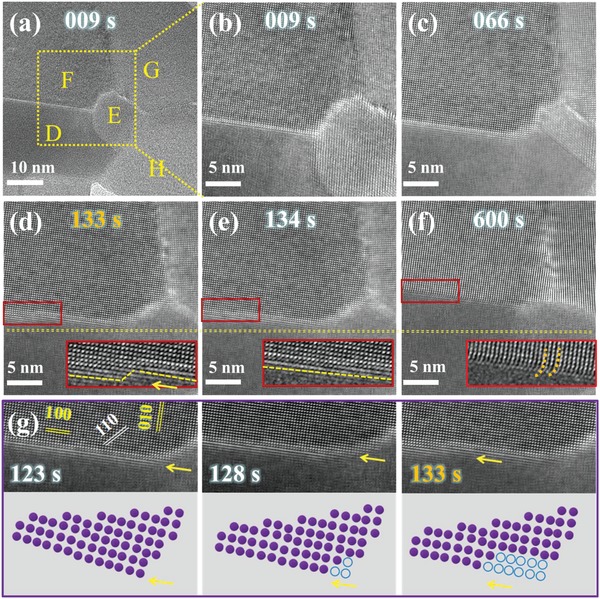
The growth process of the PbSe NCs with comparatively larger size at elevated temperature of 380 °C. a) The HRTEM image of PbSe NCs, in which the NCs D E F G and H are clearly labeled. b) The magnified image of the yellow dotted box region in inset (a). c) NC E is gradually merged into NC D after 66 s. d–f) The HRTEM images of the interfacial structures between NCs D and F during the heat induced growth process. g) The process of atomic re‐arrangements at the grain boundary just before the moment of 133 s, and the corresponding sketches of atomic re‐arrangements. c–f) HRTEM images of the same region as inset (b) at different time points.

As mentioned above, after keeping the temperature at 280 °C for some time, the growth of the NCs and migration of grain boundaries seem to stop. In order to verify the temperature dependence of the NCs growth, the temperature is increased to 380 °C, where the in situ structural variations are shown in the Movie S3 (Supporting Information) and the corresponding selected snapshots are displayed in **Figure** **4**. As the temperature increases, the NCs continue to grow into even larger size, proving that the grain boundaries intersecting NCs with larger size are more stable than those with smaller size, and higher energy is needed for further structural reconstruction.

Figure 4a–c show the process of grain boundary migration between NC D and NC E. Similar to NCs 1 and 2 in Figure 3, NC E with much smaller size merges into NC D through interfacial atom reconfiguration. It should be noted that during the growth process, the NCs with larger size play a leading role, and those with smaller size tend to transform themselves to match the bigger ones to lower their high surface energies. NCs with relatively smaller size would have higher surface energies, which is not beneficial to their structural stability.^43^, ^44^, ^45^ While smaller NCs are inclined to merge into bigger crystals, those bigger crystals also show competitive growth at the interfacial regions. Figure 4d–f focus on the growth process of bigger crystals. While the grain boundary between NCs D and F also moves through atoms reconfiguration, the reconfiguration process takes place in the first few atomic layers closest to the grain boundary, and the atoms of the same layer do not transform themselves at the same time, which could be explained by local strain distributions. As displayed in Figure 4f, different from the central region, the lattices near the interface are severely bent which indicate high levels of strain in that region. This phenomenon could be attributed to the lattice mismatch between crystals of different orientations. The geometric phase analysis (GPA) procedure compatible with Digital Micrograph is applied to Figure 4f, and the results are shown in Figure S4 (Supporting Information): the vertical strain profile (Figure S4d, Supporting Information) indeed shows abruptly increased strain level right at the grain boundary which is pushed upward during prolonged heating process.

Careful analysis of the atomic reconfiguration at the grain boundary reveals that the atomic reconfiguration is more than an overall rearrangement of atoms layer by layer. As shown in Figure 4g, the reconfiguration starts from the corner of NC F at (110) face, which should be due to the relatively high surface energy of (110) face and the high density of surface atoms, as it is reported that the surface free energies satisfy the following relation: γ_(111)_ < γ_(100)_ < γ_(110)_ for fcc structures.^46^ After 6 s, the atomic reconfiguration transfers to (100) face, which is perpendicular to the direction of grain boundary migration. Here, the distribution of strain may be expressed as the main contributor to the reconfiguration of interface atoms. It is reported that the curved boundary is expected to migrate toward its center of curvature with a velocity proportional to local radius of the curved boundary.^47^ In our case, high level of strain piles up at the grain boundary, and the different behaviors of the atoms within the same atomic layer could be attributed to the slightly different local boundary curvatures induced by strain, which leads to a different speed of boundary motion and to a sharp interface (Figure 4f). Different from the grain boundary motion between NCs D and F, the grain boundary between NCs F and G hardly moves during the whole heating process. The corresponding GPA result (Figure S4b, Supporting Information) shows that lots of dislocations accumulate at the grain boundary. Such high density of dislocations would release the local strain energy,^48^, ^49^ prevent the reconfiguration of the interface atoms, and result in a pinned grain boundary.

It is apparent that the orientation of the NCs is more random at higher temperature, and high angle grain boundaries are formed with low degree of freedom for crystal rotation, which facilitate the grain boundary migration growth mode. On the other hand, it would be interesting to check those regions of lower density of NCs with free spaces around them, and see which growth mode would they follow. It turns out that in most cases they would still follow the grain boundary migration growth mode, and one example is presented in Figure S5 in the Supporting Information. These observations indicate that besides local degree of freedom, the crystal size would also play importent part in determining the growth mode. At high temperature, the NCs have grown into larger sizes, which would prevent the crystal rotation for oriented attachment, and grain boundary migration is the prevailing growth mode in that case. In special case, we also find a region including large NCs of similar orientions and surrounding free spaces, which shows further growth following the oriented attachment growth mode at 380 °C (Movie S4 and Figure S6 in the Supporting Information). which could be attributed to the collective effects of higher temperature and the increased degree of freedom. As the NCs with random orientations merge into bigger crystals, free spaces appear around the large crystals with increased degree of freedom, and the oriented attachment growth becomes possible for large NCs of similar orientations.

## 3 Conclusion

In summary, PbSe NCs with uniform cubic morphologies are successfully prepared, and the temperature dependence of the growth process of the NCs is directly observed with in situ TEM technique. As the temperature increases from room temperature, the NCs start to grow following the oriented attachment growth mode, the contacted NCs coalesce to reduce their surface energies and rotate to decrease the interfacial lattice mismatch. At moderate temperature, the NCs are activated with the inclination to gather together, more NCs would contact with more than one NCs, due to which the rotation of the NCs are limited with lower degree of freedom, and grain boundary migration with the reconfiguration of the interfacial atoms would take place as the major growth mode. The different contact faces and high angle grain boundaries lead to random arrangements of atoms at the interfaces, the corresponding density functional theory calculations show that these interfaces are unstable with high energies, and NCs with smaller sizes are inclined to be assimilated by larger NCs. The grain boundary may also extend itself through combining adjacent grain boundaries with similar orientations. Grain boundaries intersecting larger NCs are comparatively more stable, and higher temperature is required for further crystal growth and grain boundary migration. At even higher temperature, the reconfiguration of grain boundaries between NCs of even larger size occur through strain mediated atom migrations since high levels of strain pile up at the grain boundary. Where the strain is released by high density of dislocations, the grain boundary is obviously pined without further migration. For larger‐sized NCs formed at high temperature with surrounding free spaces, grain boundary migration growth mode still prevails unless the contacing NCs are of similar orientations, which show oriented attachment locally. Thus, the whole growth process of PbSe NCs is highly dependent on growth temperature, with hybrid growth modes of oriented attachment and grain boundary migration. The results could also be applicable to NCs of similar morphologies and atomic structures.

## Conflict of Interest

The authors declare no conflict of interest.

## Supporting information

Supplementary
