# Supplementary material for: Hybrid Growth Modes of PbSe Nanocrystals with Oriented Attachment and Grain Boundary Migration
Source: Adv Sci (Weinh). 2019 Feb 28;6(9):1802202. doi: 10.1002/advs.201802202 (PMC6498134; doi:10.1002/advs.201802202)
Supplement: Supplementary file 1 — Supplementary [file ADVS-6-1802202-s001.pdf]

Copyright WILEY-VCH Verlag GmbH & Co. KGaA, 69469 Weinheim, Germany, 2019.

ADVANCED SCIENCE
Open Access

Supporting Information

for *Adv. Sci.,* DOI: 10.1002/advs.201802202

Hybrid Growth Modes of PbSe Nanocrystals with Oriented Attachment and Grain Boundary Migration

Feng Cheng, Linyuan Lian, Luying Li, Jiangyu Rao, Chen Li, Tianyu Qi, Zhi Zhang, Jianbing Zhang,* and Yihua Gao*

Supporting Information

Hybrid Growth Modes of PbSe Nanocrystals with Oriented Attachment and Grain Boundary Migration

Feng Cheng, Linyuan Lian, Luying Li*, Jiangyu Rao, Chen Li, Tianyu Qi, Zhi Zhang, Jianbing Zhang*, Yihua Gao

Feng Cheng, Prof. Luying Li, Jiangyu Rao, Chen Li, Tianyu Qi
Center for Nanoscale Characterization & Devices
Wuhan National Laboratory for Optoelectronics
Huazhong University of Science and Technology, 1037 Luoyu Road, Wuhan, Hubei 430074, China
E-mail: luying.li@hust.edu.cn.

Linyuan Lian, Prof. Jianbing Zhang
Engineering Research Center for Functional Ceramics, Ministry of Education
School of Optical and Electronic Information
Huazhong University of Science and Technology, 1037 Luoyu Road, Wuhan, Hubei 430074, China
E-mail: jbzhang@hust.edu.cn

Prof. Zhi Zhang, Prof. Yihua Gao
School of Physics
Huazhong University of Science and Technology, 1037 Luoyu Road, Wuhan, Hubei 430074, China

1. Synthesis of PbSe nanocrystals

2. Purification of PbSe nanocrystals

3. DFT calculations

4. Analysis of grain boundary migration and integration

5. Analysis of dislocation and strain

6. Growth process of PbSe nanocrystals of larger size

1. Synthesis of PbSe nanocrystals

Chemicals. $PbCl_2$ (99.999%), oleic acid (OA, tech. grade, 90%), 1-octadecene (ODE, tech. grade, 90%), trioctylphosphine (TOP, tech. grade, 90%) were purchased from Alfa Aesar. Oleylamine (OLA, tech. grade, 70%), Diphenylphosphine (DPP, 95%), selenium powder (99.999%) were purchased from Aladdin. Tetrachloroethylene (TCE, ≥98.5%), hexane (≥97%), and acetone (≥99.5%) were purchased from Sinopharm Chemical Reagent. All the chemicals were used as received. TOPSe and DPPSe (selenium powder dissolved in TOP and DPP respectively) were used as the molecular precursors. DPPSe was made by heating the DPP and Se powder at 120 °C for 30 min.

The PbSe nanocrystals were synthesized via an adaptation of a reported approach developed by Zhang et al.[1] In a typical synthesis, OLA and 9 mmol of $PbCl_2$ were degassed under vacuum at 80 °C and heated to 140 °C under nitrogen, the temperature was maintained for 30 min until a white and turbid solution was obtained. Then the suspension was allowed to cool to 30 °C, 110 uL of 1M DPPSe diluted in 2 mL of ODE and 0.57 mL of 1M TOPSe were injected sequentially to the lead precursor solution at 30 °C. Next, the reaction solution was reheated to 160 °C and maintained at 160 °C for 15 min under vigorous stirring for the growth of the nanocrystals. After that, 1.14 mL of 1M TOPSe and 220 uL of DPP diluted in 2 mL of ODE were injected sequentially to the growth solution, and the solution was maintained at 160 °C for another 15 min. The crude solution containing PbSe nanocrystals was obtained.

2. **Purification of PbSe nanocrystals.**

The crude solution was quenched by a water bath, 20 mL of hexane and 20 mL of OA were added at 70 °C and 40 °C, respectively, followed by vigorous stirring for 10 min. The unreacted lead precursors and PbSe nanocrystals were precipitated from the raw solution by the first centrifugation, and the supernatant was discarded. Then 10 mL of tetrachloroethylene was added to the precipitates to disperse, after the second centrifugation, the supernatant contained PbSe nanocrystals and the precipitate was discarded. The supernatant was collected and precipitated by adding acetone. After the third centrifugation, the supernatant was discarded and the precipitated PbSe nanocrystals were collected.

3. DFT calculations

Density functional theory (DFT) calculations were performed using the Vienna Ab-initio Simulation Package (VASP)[2]. The interactions between core ions and valence electrons were described by the projector augmented wave method (PAW).[3] The exchange correlation part was described with the spin-polarized generalized gradient approximations (GGA) in the scheme of Perdew, Burke, Ernzerhof (PBE).[4] An energy cutoff of 500 eV was used with Monkhorst k-points[5] sampling scheme 5×1×1 for geometry optimization, while a larger grid 9×1×1 was used for electronic structure computation. Conjugated gradient method was used to the geometry optimization, all of the atomic coordinates were fully relaxed until the maximal force on each atom was less than 0.02 eV/Å, and the convergence condition for energy was

10^{-4} eV. The diffusion pathway of dislocations and the corresponding energy barrier were optimized with nudged elastic band (NEB) method.[6]

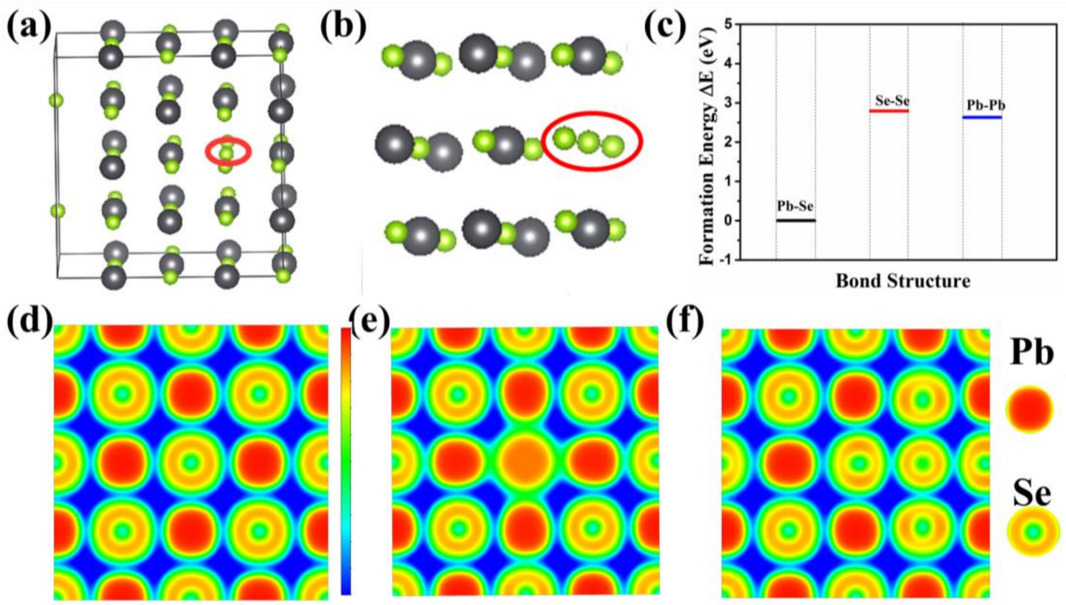

Figure S1. The formation energy and accumulated charge of Pb-Pb and Se-Se bonds. (a-b) The atomic models of PbSe with atom substitution, based on which the Se-Se and Pb-Pb bonds are formatted. (c) The formation energies of Pb-Se, Se-Se and Pb-Pb bonds, and the energy of Pb-Se is set to be zero as reference. (d-f) The accumulated charges of the material with Pb-Se, Pb-Pb and Se-Se bonds, respectively.

The determination of formation energies of Pb-Pb and Se-Se bonds are further conducted by first-principle calculations to rationalize the corresponding structural stability. The formation energy is defined as follows: $E_f = E_{final} - E_{intial} + \Sigma n_i\mu_i$, where E_{final} and E_{intial} are total energies of the defective and ideal structures, respectively. n_i and μ_i are the number and chemical potential of the atoms added to ($n_i > 0$) or take from ($n_i < 0$) the ideal unit cell to create the defects.[7,8] The chemical potentials of Pb

and Se atoms are the total energies of Pb and Se atoms. The resultant formation energies of Pb-Pb and Se-Se are as high as 2.79 and 2.63 eV, respectively. The high formation energies indicate that the Pb-Pb and Se-Se bonds are unstable. Meanwhile, the electronic localization function (ELF) also shows that the Pb-Se bond is highly ionic. The accumulated charges around Pb-Pb and Se-Se bonds suggest weakened covalence. Thus, the structure stability is reduced with the formation of Pb-Pb and Se-Se bonds.

4. Analysis of grain boundary migration and integration

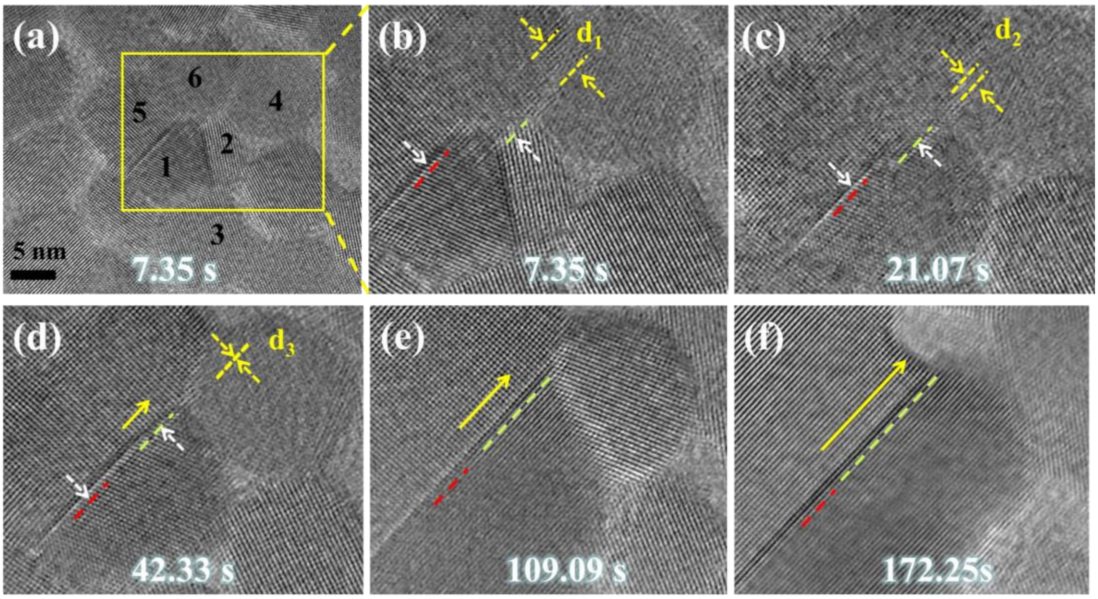

Figure S2. The grain boundary migration and integration of PbSe nanocrystals (NCs). (a-c) The migration of the grain boundary between NCs 1 and 5, during which the distance d between the grain boundary of NCs 1-5 and that of NCs 2-6 is decreased significantly. (d) The integration of the grain boundaries of NCs 1-5 and NCs 2-6. (e-f) The extension and further migration of the grain boundaries.

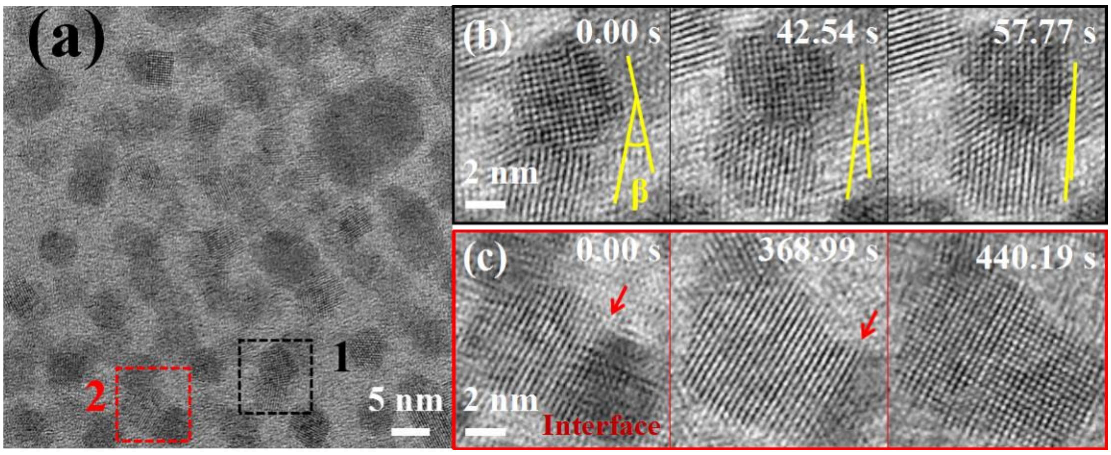

Figure S3. The growth process of PbSe NCs with smaller size of 5 nm heated at 180 °C, during which the NCs grow following hybrid growth modes of oriented attachment and grain boundary migration. (a) TEM image of the PbSe NCs. (b) The growth of two NCs with high degree of freedom (black box region in (a)) following oriented attachment growth mode. (c) The growth of several PbSe NCs with low degree of freedom (red box region in (a)) following grain boundary migration growth mode.

5. **Analysis of dislocation and strain**

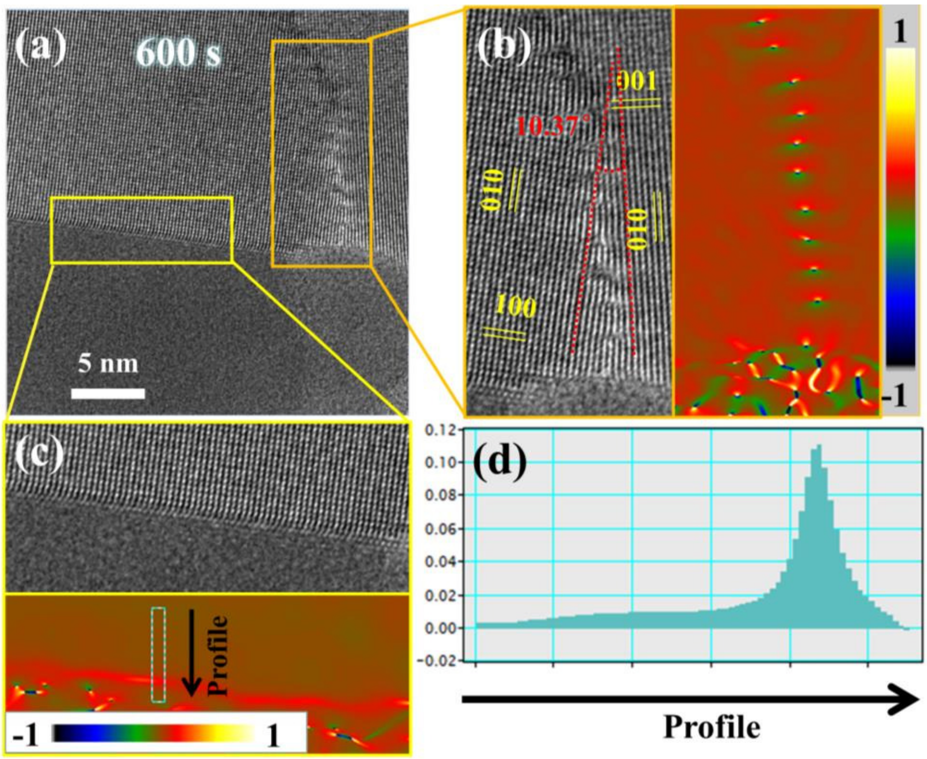

Figure S4. Analysis of dislocation and strain. (a) The HRTEM image of the PbSe NCs corresponding to Figure 4d. (b) The magnified image of the brown box region in (a) and the corresponding geometric phase analysis (GPA) on the right hand side showing distribution of serial dislocations. (c) The HRTEM image and GPA of the grain boundary corresponding to the yellow box region in (a). (d) The strain line profile corresponding to the green box region in (c).

6. **Growth process of PbSe nanocrystals of larger size at elevated temperature**

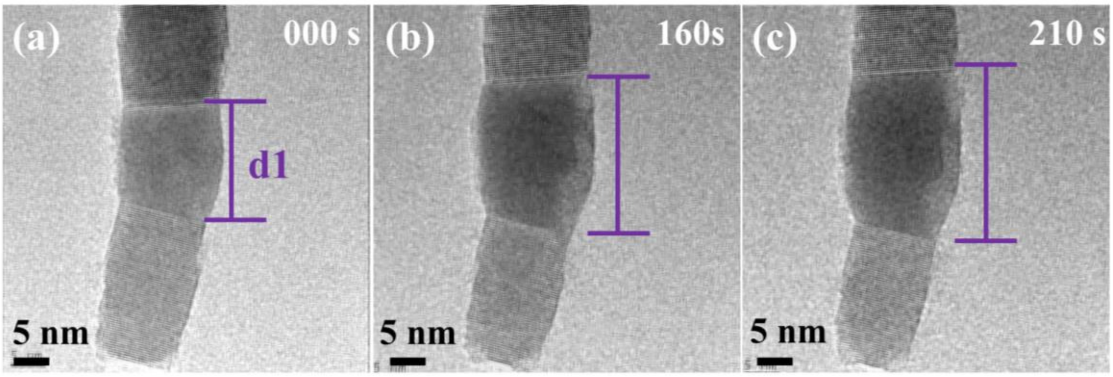

Figure S5. The growth process of PbSe NCs with comparatively larger size and high degree of freedom at elevated temperature of 380 °C, which show obvious grain boundary migration with prolonged time.

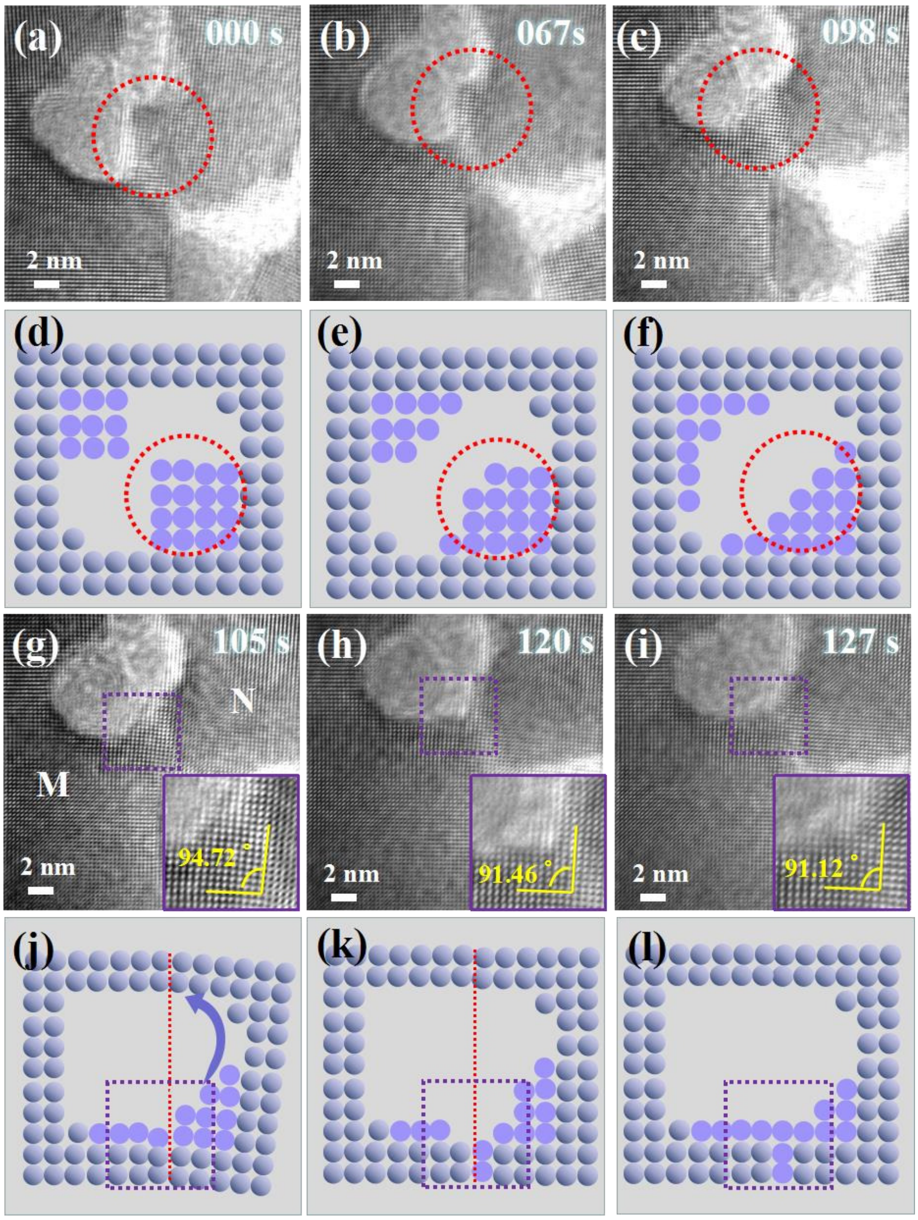

Figure S6. HRTEM images showing the growth process of PbSe nanocrystals of larger size with surrounding vacuum regions. (a-c) The migration of the surface atoms. (d-f) The sketches showing surface atom migrations corresponding to (a-c). (g-i) The heat-induced growth of PbSe nanocrystals following oriented attachment growth mode, during which the nanocrystals on both sides of the grain boundary rotate to match up with each other through surface atom reconstructions. (j-l) The sketches illustrating the oriented attachment growth process corresponding to (g-i).

Reference

[1] L. Lian, Y. Xia, C. Zhang, B. Xu, L. Yang, H. Liu, D. Zhang, K. Wang, J. Gao, J. Zhang, Chem. Mater. **2018**, 30 (3), 982.

[2] G. Kresse, J. Hafner, Phy. Rev. B **1994**, 49 (20), 14251.

[3] P. E. Blöchl, Phy. Rev. B **1994**, 50 (24), 17953.

[4] J. P. Perdew, K. Burke, M. Ernzerhof, Phy. Rev. Lett. **1996**, 77 (18): 3865.

[5] H. J. Monkhorst, J. D. Pack, Phy. Rev. B **1976**, 13 (12), 5188.

[6] G. Henkelman, H. J. Jónsson, Chem. Phy. **2000**, 113 (22), 9978.

[7] J. Wang, J. Liu, B. Zhang, H. Wan, Z. Li, X. Ji, K. Xu, C. Chen, D. Zha, L. Miao, J. Jiang, Nano Energy **2017**, 42, 98.

[8] J. Wang, J. Liu, B. Zhang, F. Cheng, Y. Ruan, X. Ji, K. Xu, Chen, C. L. Miao, J. Jiang, Nano Energy **2018**, 53, 144.
